# Fragmented implementation of maternal and child health home-based records in Vietnam: need for integration

**DOI:** 10.3402/gha.v9.29924

**Published:** 2016-02-25

**Authors:** Hirotsugu Aiga, Vinh Duc Nguyen, Cuong Dinh Nguyen, Tho Thi Thi Nguyen, Lien Thi Phuong Nguyen

**Affiliations:** 1 Human Development Department, Japan International Cooperation Agency, Tokyo, Japan; 2 Department of Global Health, Milken Institute School of Public Health, George Washington University, Washington, DC, USA; 3 Maternal and Child Health Department, Ministry of Health, Hanoi, Vietnam; 4 Sustainable Health Development Center, VietHealth, Hanoi, Vietnam; 5 Department of Community Health and Preventive Medicine Network Coordination, National Institute of Hygiene and Epidemiology, Hanoi, Vietnam

**Keywords:** maternal, newborn, and child health, home-based records, maternal and child health handbook, child vaccination card, health information systems

## Abstract

**Background:**

Home-based records (HBRs) are globally implemented as the effective tools that encourage pregnant women and mothers to timely and adequately utilise maternal and child health (MCH) services. While availability and utilisation of nationally representative HBRs have been assessed in several earlier studies, the reality of a number of HBRs subnationally implemented in a less coordinated manner has been neither reported nor analysed.

**Objectives:**

This study is aimed at estimating the prevalence of HBRs for MCH and the level of fragmentation of and overlapping between different HBRs for MCH in Vietnam. The study further attempts to identify health workers’ and mothers’ perceptions towards HBR operations and utilisations.

**Design:**

A self-administered questionnaire was sent to the provincial health departments of 28 selected provinces. A copy of each HBR available was collected from them. A total of 20 semi-structured interviews with health workers and mothers were conducted at rural communities in four of 28 selected provinces.

**Results:**

Whereas HBRs developed exclusively for maternal health and exclusively for child health were available in four provinces (14%) and in 28 provinces (100%), respectively, those for both maternal health and child health were available in nine provinces (32%). The mean number of HBRs in 28 provinces (=5.75) indicates over-availability of HBRs. All 119 minimum required items for recording found in three different HBRs under nationwide scale-up were also included in the *Maternal and Child Health Handbook* being piloted for nationwide scaling-up. Implementation of multiple HBRs is likely to confuse not only health workers by requiring them to record the same data on several HBRs but also mothers about which HBR they should refer to and rely on at home.

**Conclusions:**

To enable both health workers and pregnant women to focus on only one type of HBR, province-specific HBRs for maternal and/or child health need to be nationally standardised. Moreover, to ensure a continuum of maternal, newborn, and child health care, the HBRs currently fragmented into different MCH stages (i.e. pregnancy, delivery, child immunisation, child growth, and child development) should be integrated. Standardisation and integration of HBRs will help increase technical efficiency and financial sustainability of HBR operations.

## Background

Home-based records (HBRs) for maternal and child health (MCH) have been operationalised as an essential part of national MCH programmes in both developing and developed countries. They play an important role in documenting the results of antenatal check-ups, delivery, postnatal check-ups, child immunisation, child growth and child development, and in keeping them available at home (1). The most commonly implemented HBR is child vaccination card, which serves as a self-monitoring tool for national child immunisation programmes and also as a reliable data source for estimating child immunisation coverage (2). A primary purpose of child vaccination card is to foster continuity of immunisation service delivery and help facilitate communication between health workers and children's mothers and caregivers (3). Earlier studies reported on the effectiveness of child vaccination card in increasing the immunisation completion rate (3, 4). Another commonly implemented HBR is growth chart, which has been implemented in over 150 countries (5). A previous study identified no significant differences in change in weight for age between children whose mothers understood the chart and those who did not (6). There is also pregnancy care card, which focuses only on recording the results of antenatal care and delivery (7). Some countries implement maternal and child health handbook, an integrated HBR for both a mother and her child applicable to all stages of maternal, newborn, and child health throughout pregnancy, delivery, birth, postnatal period, infancy, and childhood (8).

It is a reality that in many countries multiple different HBRs for MCH are existing, available, and implemented, often in a fragmented manner without integration, standardisation, or coordination. Parallel operations of multiple HBRs can be attributed to several ongoing standalone MCH-related vertical programmes and less controllable *ad hoc* implementation of province-specific HBRs at the subnational level. Several previous studies conducted international comparisons of the prevalence of nationally representative child vaccination cards between countries (2, 9, 10). Yet, there are extremely few earlier studies that compared the prevalence of HBRs between provinces and between MCH-related vertical programmes within a country or that further measured the levels of fragmentation and overlapping among those multiple HBRs.

Vietnam, one of the eight countries on track to meet both Millennium Development Goals 4 and 5, has made rapid progress in reducing maternal mortality ratio by 78%, from 233 per 100,000 live births in 1990 to 49 in 2013, and under-five mortality rate by 53%, from 51 per 1,000 live births in 1990 to 24 in 2013 (11). HBRs for MCH are likely to have contributed to reductions in these mortality rates, by promoting timely and adequate utilisation of maternal, newborn, and child health services (12). Yet, there were reportedly a number of different HBRs being implemented in parallel at different levels in different parts of the country for different MCH-related vertical programmes. Nevertheless, no previous studies described the reality of fragmented and duplicated operations of HBRs at the national and subnational levels in the country.

This study is aimed at estimating the prevalence of HBRs for MCH and the level of fragmentation of and overlapping between different HBRs for MCH, in Vietnam. This study further attempts to identify health workers’ perceptions of HBR operations and mothers’ perceptions of HBR utilisations.

## Methods

### Study areas

In Vietnam, the 64 provinces are grouped into six administrative regions according to their geographic locations. From each administrative region, four representative provinces were selected by applying three criteria. First, to ensure socio-demographic diversity, two or three of the four provinces in each administrative region were selected because they had a greater rural population than the regional mean, whereas the other provinces chosen had greater urban populations than the regional mean. Second, to ensure geographic diversity, all five types of provinces (coastal, mountainous, mountainous and coastal, highland, and plain) were selected (Table 1). In addition to these 24 provinces (6 regions×4 provinces/region), four provinces were selected (Dien Bien, Hoa Binh, Thanh Hoa, and An Giang) where the standardised MCH handbook (*MCH Handbook #1*; Table 1) was piloted by the Vietnamese Ministry of Health (MoH) and the Japan International Cooperation Agency (JICA). Thus, a total of 28 (24 + 4) provinces were selected as the study areas.

**Table 1:** Criteria for province selection and prevalence of MCH home-based records in 28 selected provinces

	Selection criteria			Maternal and/or child health home-based records implemented in selected 28 provinces																							
		
				[1]	[2]	[3]	[4]	[5]	[6]	[7]	[8]	[9]	[10]	[11]	[12]	[13]	[14]	[15]	[16]	[17]	[18]	[19]	[20]	[21]	[22]	[23]	
					
Region and province	Piloting standardized MCH Handbook1	Urban or rural^a^	Geographic characteristics^b^	Antenatal Care & Monitoring Handbook	Antenatal Care Card	Pregnancy Monitoring Card	Child Health Monitoring Handbook	Child Growth Monitoring Chart	Vaccination Card	Vaccination Handbook #1^c^	Vaccination Handbook #2^c^	Vaccination Handbook for Children	MCH^g^ Handbook #1^d^	MCH^g^ Handbook #2^d^	MCH^g^ Handbook #3^d^	MCH^g^ Handbook #4^d^	MCH^g^ Monitoring Card at Home #1^e^	MCH^g^ Monitoring Card at Home #2^e^	MCH^g^ Monitoring Card at Home #3^e^	MNH^h^ Monitoring Card at Home #1^f^	MNH^h^ Monitoring Card at Home #2^f^	MNH^h^ Monitoring Card at Home #3^f^	MNH^h^ Monitoring Card at Home #4^f^	MNH^h^ Monitoring Card at Home #5^f^	Nutrition & health Care Handbook	Health Checkup Handbook	Total number of HBRs being implemented
Target groups^i^				**M**	**M**	**M**	**M**	**C**	**C**	**C**	**MC**	**C**	**MC**	**MC**	**MC**	**MC**	**MC**	**MC**	**MC**	**MC**	**MC**	**MC**	**MC**	**MC**	**C**	**A**	
*Red River Delta Rgn*																											
Thai Binh		**R**	**Ct**					**X**	**X**	**X**															**X**	**X**	**5**
Nam Dinh		**R**	**Ct**					**X**	**X**	**X**															**X**	**X**	**5**
Hai Phong		**U**	**Ct**					**X**	**X**	**X**															**X**	**X**	**5**
Quang Ninh		**U**	**Ct**					**X**	**X**	**X**		**X**													**X**	**X**	**6**
*Northern Midlands & Mountain Rgn*																											
Dien Bien	**X**	**R**	**Mt**					**X**	**X**	**X**			**X**												**X**	**X**	**6**
Hoa Binh	**X**	**R**	**Mt**			**X**		**X**	**X**	**X**			**X**										**X**		**X**	**X**	**8**
Cao Bang		**U**	**Mt**					**X**	**X**	**X**				**X**											**X**	**X**	**6**
Ha Giang		**R**	**Mt**					**X**	**X**	**X**															**X**	**X**	**5**
Thai Nguyen		**U**	**Mt**					**X**	**X**	**X**															**X**	**X**	**5**
Yen Bai		**U**	**Mt**					**X**	**X**	**X**															**X**	**X**	**5**
*North Central & Central Coastal Rgn*																											
Thanh Hoa	**X**	**R**	**Mt-Ct**					**X**	**X**	**X**			**X**						**X**		**X**				**X**	**X**	**8**
Quang Binh		**R**	**Mt-Ct**					**X**	**X**	**X**															**X**	**X**	**5**
Ha Tinh		**R**	**Mt-Ct**					**X**	**X**	**X**															**X**	**X**	**5**
Binh Thuan		**U**	**Mt-Ct**					**X**	**X**	**X**								**X**							**X**	**X**	**6**
Quang Nam		**U**	**Mt-Ct**					**X**	**X**	**X**												**X**			**X**	**X**	**6**
*Central Highlands Rgn*																											
Lam Dong		**U**	**HL**					**X**	**X**	**X**															**X**	**X**	**5**
Dak Lak		**R**	**HL**					**X**	**X**	**X**															**X**	**X**	**5**
Kon Tum		**U**	**HL**					**X**	**X**	**X**															**X**	**X**	**5**
Gia Lai		**R**	**HL**					**X**	**X**	**X**															**X**	**X**	**5**
*South East Rgn*																											
Tay Ninh		**R**	**Pl**					**X**	**X**	**X**															**X**	**X**	**5**
Binh Duong		**U**	**Pl**					**X**	**X**	**X**															**X**	**X**	**5**
Ba Ria - Vung Tua		**U**	**Pl**					**X**	**X**	**X**														**X**	**X**	**X**	**6**
Binh Phuoc		**R**	**Pl**	**X**			**X**	**X**	**X**	**X**															**X**	**X**	**7**
*Mekong River delta Rgn*																											
An Giang	**X**	**U**	**Pl**		**X** **j**			**X****j**	**X****j**	**X****j**	**X****j**		**X**		**X****j**		**X****j**			**X** **j**,**k**					**X****j**	**X****j**	**11**
Ben Tre		**R**	**Ct**					**X**	**X**	**X**						**X**									**X**	**X**	**6**
Dong Thap		**R**	**Pl**					**X**	**X**	**X**															**X**	**X**	**5**
Can Tho		**U**	**Pl**					**X**	**X**	**X**															**X**	**X**	**5**
Long An		**R**	**Pl**					**X**	**X**	**X**															**X**	**X**	**5**

**Total**	**4**	**15**	**13**	**1**	**1**	**1**	**1**	**28**	**28**	**28**	**1**	**1**	**4**	**1**	**1**	**1**	**1**	**1**	**1**	**1**	**1**	**1**	**1**	**1**	**28**	**28**	

a R: Rural dominant province with a greater proportion of rural populations than the regional mean, U: Urban dominant province with a greater proportion of urban populations than the regional mean.

b Ct: Costal province, HL: Highland province, Mt: Mountenous province, Mt-Ct: Mountenous & costal province, Pl: Plain province.

c,d,e,f To distinguish each from these home-based records titled the same, numbering was made by the authors such as #1, #2, #3, #4 and #5.

g MCH: Maternal and Child Health.

h MNH: Maternal and Newborn Health.

i M: Pregnant women / mothers, C: Children, MC: Pregnant women / mothers and children, A: All age groups of both sexes.

j In An Giang province, though 11 home-based records were available, recently only “MCH Handbook #1” has been implemented by temporarily suspending the use of others.

k Available only in Phu Tan district of An Giang Province.

**Table 2:** Number of recording items on respective technical topics found in MCH home-based records

					The number of maternal and/or child health home-based records with respective recording topics^a^																			
					
				*Maternal health care*	Pregnancy							Delivery					Postnatal (mother)							
					
					Antenatal care visit ≥4 times	Tetanus immunization	PMTCT of HIV	Danger signs in pregnancy	Community behavior	Others	Total	Skilled obstetric care	PMTCT of HIV	Emergency obstetric care	Clean delivery education	Total	Healthy bahavior for mothers	Family planning	Water; sanitation & hygiene	Danger signs & care seeking	Others	Total	–	–
					
				*Child health care*								Birth					Postnatal (newborn)							
												
*#*	Title of home-based records for maternal and child health	Publication year	Publisher		–	–	–	–	–	–	–	Essential care for neonate	PMTCT of HIV	Emergency newborn care	Early neonate care education	Total	Neonate illness management	Care for preterm newborns	Care for HIV+ newborns	Healthy home behavior	Water; sanitation & hygiene	Management of malnutrition	Others	Total
[1]	Antenatal Care & Monitoring Handbook	2013	Binh Phuoc provincial DoH	*Maternal*	111	4	1	156	0	31	303	0	0	0	0	0	0	0	0	0	0	0	–	–
				*Child*	–	–	–	–	–	–	–	0	0	0	0	0	0	0	0	0	0	0	0	0
[2]	Antenatal Care Card	[n.a.]	An Gian provincial DoH	*Maternal*	44	4	1	50	0	25	124	0	0	0	0	0	0	0	0	0	0	0	–	–
				*Child*	–	–	–	–	–	–	–	0	0	0	0	0	0	0	0	0	0	0	0	0
[3]	Pregnancy Monitoring Card	[n.a.]	Hoa Binh provincial DoH	*Maternal*	20	0	0	54	0	15	89	0	0	0	0	0	0	0	0	0	0	0	–	–
				*Child*	–	–	–	–	–	–	–	0	0	0	0	0	0	0	0	0	0	0	0	0
[4]	Child Health Monitoring Handbook	[n.a.]	Binh Phuouc provincial DoH	*Maternal*	0	0	0	0	0	3	3	3	0	1	0	4	0	0	0	0	0	0	–	–
				*Child*	–	–	–	–	–	–	–	5	0	0	0	5	0	0	0	0	0	0	0	0
[5]	Child Growth Monitoring Chart	2013	National Institute of Nutrition, MoH	*Maternal*	0	0	0	0	0	0	0	0	0	0	0	0	0	0	0	0	0	0	–	–
				*Child*	–	–	–	–	–	–	–	0	0	0	0	0	0	0	0	0	0	0	0	0
[6]	Vaccination Card	2013	National Institute of Hygiene & Epidemiology, MoH	*Maternal*	0	0	0	0	0	0	0	0	0	0	0	0	0	0	0	0	0	0	–	–
				*Child*	–	–	–	–	–	–	–	0	0	0	0	0	0	0	0	0	0	0	0	0
[7]	Vaccination Handbook #1^d^	2013	National Institute of Hygiene & Epidemiology, MoH	*Maternal*	0	0	0	0	0	2	2	0	0	0	0	0	0	0	0	0	0	0	–	–
				*Child*	–	–	–	–	–	–	–	0	0	0	0	0	0	0	0	0	0	0	0	0
[8]	Vaccination Handbook #2^d^	2013	An Gian provincial DoH	*Maternal*	8	20	0	0	0	0	28	0	0	0	0	0	0	0	0	0	0	0	–	–
				*Child*	–	–	–	–	–	–	–	0	0	0	0	0	0	0	0	0	0	0	0	0
[9]	Vaccination Handbook for Children	2010	Hanoi provincial DoH	*Maternal*	0	0	0	0	0	0	0	0	0	0	0	0	0	0	0	0	0	0	–	–
				*Child*	–	–	–	–	–	–	–	0	0	0	0	0	0	0	0	0	0	0	0	0
[10]	MCH^b^ Handbook #1^e^	2013	Dept of MCH, MoH	*Maternal*	74	12	0	88	0	33	207	20	0	1	0	21	4	1	0	8	29	42	–	–
				*Child*	–	–	–	–	–	–	–	25	0	1	2	28	11	0	0	21	0	4	9	45
[11]	MCH^b^ Handbook #2^e^	[n.a.]	[n.a.]	*Maternal*	42	3	0	72	0	24	141	13	0	2	0	15	4	1	0	8	20	33	–	–
				*Child*	–	–	–	–	–	–	–	17	0	2	1	20	7	0	0	17	0	4	14	42
[12]	MCH^b^ Handbook #3^e^	2010	An Gian provincial DoH	*Maternal*	110	3	0	180	0	24	317	4	0	2	0	6	1	0	0	5	7	13	–	–
				*Child*	–	–	–	–	–	–	–	10	0	1	1	12	0	0	0	4	0	0	0	4
[13]	MCH^b^ Handbook #4^e^	2013	Be Tre provincial DoH	*Maternal*	62	3	0	100	0	20	185	4	0	2	0	6	1	0	0	5	7	13	–	–
				*Child*	–	–	–	–	–	–	–	10	0	1	1	12	0	0	0	4		0	0	4
[14]	MCH^b^ Monitoring Card at Home #1^f^	2008	An Gian provincial DoH	*Maternal*	32	0	0	45	0	17	94	7	0	2	0	9	1	225	0	8	7	241	–	–
				*Child*	–	–	–	–	–	–	–	7	0	0	2	9	0	0	0	0		0	0	0
[15]	MCH^b^ Monitoring Card at Home #2^f^	2013	Binh Thuan provincial DoH	*Maternal*	20	0	0	45	0	19	84	7	0	2	0	9	1	225	0	8	7	241	–	–
				*Child*	–	–	–	–	–	–	–	7	0	0	2	9	0	0	0	0		0	0	0
[16]	MCH^b^ Monitoring Card at Home #3^f^	2013	Thanh Hoa provincial DoH	*Maternal*	32	6	0	47	0	19	104	7	0	2	0	9	1	225	0	8	7	241	–	–
				*Child*	–	–	–	–	–	–	–	7	0	0	2	9	0	0	0	0		0	0	0
[17]	MNH^c^ Monitoring Card at Home #1^g^	[n.a.]	Phu Tan district, An Giang Province	*Maternal*	23	6	0	29	0	16	74	5	0	2	0	7	2	225	0	6	4	237	–	–
				*Child*	–	–	–	–	–	–	–	6	0	0	0	6	0	0	0	0		0	0	0
[18]	MNH^c^ Monitoring Card at Home #2^g^	2013	Thanh Hoa provincial DoH	*Maternal*	23	6	0	29	0	16	74	5	0	2	0	7	2	225	0	6	4	237	–	–
				*Child*	–	–	–	–	–	–	–	6	0	0	0	6	0	0	0	0		0	0	0
[19]	MNH^c^ Monitoring Card at Home #3^g^	2013	Quang Nam provinical DoH	*Maternal*	29	8	0	45	0	16	98	7	0	2	0	9	1	225	0	8	7	241	–	–
				*Child*	–	–	–	–	–	–	–	7	0	0	2	9	0	0	0	0		0	0	0
[20]	MNH^c^ Monitoring Card at Home #4^g^	2013	Hoa Binh provincial DoH	*Maternal*	23	6	0	29	0	16	74	5	0	2	0	7	2	225	0	6	4	237	–	–
				*Child*	–	–	–	–	–	–	–	6	0	0	0	6	0	0	0	0		0	0	0
[21]	MNH^c^ Monitoring Card at Home #5^g^	2013	Ba Ria -Vung Tau provincial DoH	*Maternal*	56	0	0	81	0	18	155	7	0	2	0	9	1	225	0	4	3	233	–	–
				*Child*	–	–	–	–	–	–	–	7	0	0	2	9	0	0	0	0	0	0	0	0
[22]	Nutrition & Health Care Handbook	2013	National Institute of Nutrition, MoH	*Maternal*	0	0	0	0	0	0	0	1	0	0	0	1	0	0	0	0	0	0	–	–
				*Child*	–	–	–	–	–	–	–	2	0	0	0	2	0	0	0	0	0	0	0	0
[23]	Health Checkup Handbook	2013	Dept of Medical Services Administration, MoH	*Maternal*	0	0	0	0	0	0	0	0	0	0	0	0	0	0	0	0	0	0	–	–
				*Child*	–	–	–	–	–	–	–	0	0	0	0	0	0	0	0	0	0	0	0	0

**Total****h**				**Maternal**	**709**	**81**	**2**	**1050**	**0**	**314**	**2156**	**95**	**0**	**24**	**0**	**119**	**21**	**1802**	**0**	**80**	**106**	**2009**	–	–
				**Child**	**–**	**–**	**–**	**–**	**–**	**–**	**–**	**122**	**0**	**5**	**15**	**142**	**18**	**0**	**0**	**46**	**0**	**8**	**23**	**95**

**Table:** 

					The number of maternal and/or child health home-based records with respective recording topics^a^															Number of recoding items	
					
				*Maternal health care*	Maternal health																
							
					General maternal checkups	–	–	–	–	–	–	–	–	–	–	–	–	–		Maternal health care / child health care	Grand total
					
				*Child health care*	Infancy								Childhood								
							
#	Title of home-based records for maternal and child health	Publication year	Publisher		Infant illness management	Care for preterm newborns	Care for HIV+ newborns	Healthy home behavior	Water; sanitation & hygiene	Management of malnutrition	Others	Total	Child vaccination	Malaria control	Management of malnutrition	Care for HIV+ newborns	Childhood illness management	Others	Total		
[1]	Antenatal Care & Monitoring Handbook	2013	Binh Phuoc provincial DoH	*Maternal*	0	–	–	–	–	–	–	–	–	–	–	–	–	–	–	**303** (100%)	**303**
				*Child*	0	0	0	0	0	0	0	0	0	0	0	0	0	0	0	**0** (0%)	
[2]	Antenatal Care Card	[n.a.]	An Gian provincial DoH	*Maternal*	0	–	–	–	–	–	–	–	–	–	–	–	–	–	–	**124** (100%)	**124**
				*Child*	0	0	0	0	0	0	0	0	0	0	0	0	0	0	0	**0** (0%)	
[3]	Pregnancy Monitoring Card	[n.a.]	Hoa Binh provincial DoH	*Maternal*	0	–	–	–	–	–	–	–	–	–	–	–	–	–	–	**89** (100%)	**89**
				*Child*	0	0	0	0	0	0	0	0	0	0	0	0	0	0	0	**0** (0%)	
[4]	Child Health Monitoring Handbook	[n.a.]	Binh Phuouc provincial DoH	*Maternal*	0	–	–	–	–	–	–	–	–	–	–	–	–	–	–	**7** (2%)	**439**
				*Child*	0	0	0	0	0	0	0	0	200	0	124	0	66	37	427	**432** (98%)	
[5]	Child Growth Monitoring Chart	2013	National Institute of Nutrition, MoH	*Maternal*	0	–	–	–	–	–	–	–	–	–	–	–	–	–	–	**0** (0%)	**4**
				*Child*	0	0	0	0	0	0	0	0	0	0	4	0	0	0	4	**4** (100%)	
[6]	Vaccination Card	2013	National Institute of Hygiene & Epidemiology, MoH	*Maternal*	0	–	–	–	–	–	–	–	–	–	–	–	–	–	–	**0** (0%)	**17**
				*Child*	0	0	0	0	0	0	0	0	17	0	0	0	0	0	17	**17** (100%)	
[7]	Vaccination Handbook #1^d^	2013	National Institute of Hygiene & Epidemiology, MoH	*Maternal*	0	–	–	–	–	–	–	–	–	–	–	–	–	–	–	**2** (4%)	**54**
				*Child*	0	0	0	0	0	0	0	0	38	0	12	0	0	2	52	**52** (96%)	
[8]	Vaccination Handbook #2^d^	2013	An Gian provincial DoH	*Maternal*	0	–	–	–	–	–	–	–	–	–	–	–	–	–	–	**28** (41%)	**69**
				*Child*	0	0	0	0	0	0	0	0	39	0	2	0	0	0	41	**41** (59%)	
[9]	Vaccination Handbook for Children	2010	Hanoi provincial DoH	*Maternal*	0	–	–	–	–	–	–	–	–	–	–	–	–	–	–	**0** (0%)	**295**
				*Child*	0	0	0	0	0	0	0	0	295	0	0	0	0	0	295	**295** (100%)	
[10]	MCH^b^ Handbook #1^e^	2013	Dept of MCH, MoH	*Maternal*	40	–	–	–	–	–	–	–	–	–	–	–	–	–	–	**310** (36%)	**854**
				*Child*	0	0	0	12	0	16	78	106	74	0	20	0	50	221	365	**544** (64%)	
[11]	MCH^b^ Handbook #2^e^	[n.a.]	[n.a.]	*Maternal*	0	–	–	–	–	–	–	–	–	–	–	–	–	–	–	**189** (36%)	**529**
				*Child*	8	0	0	8	0	16	83	115	12	0	18	0	18	115	163	**340** (64%)	
[12]	MCH^b^ Handbook #3^e^	2010	An Gian provincial DoH	*Maternal*	0	–	–	–	–	–	–	–	–	–	–	–	–	–	–	**336** (46%)	**737**
				*Child*	1	0	0	5	0	12	46	64	33	0	12	0	90	186	321	**401** (54%)	
[13]	MCH^b^ Handbook #4^e^	2013	Be Tre provincial DoH	*Maternal*	0	–	–	–	–	–	–	–	–	–	–	–	–	–	–	**204** (37%)	**545**
				*Child*	1	0	0	5	0	12	46	64	33	0	12	0	60	156	261	**341** (63%)	
[14]	MCH^b^ Monitoring Card at Home #1^f^	2008	An Gian provincial DoH	*Maternal*	32	–	–	–	–	–	–	–	–	–	–	–	–	–	–	**376** (94%)	**398**
				*Child*	0	0	0	0	0	0	0	0	12	0	1	0	0	0	13	**22** (6%)	
[15]	MCH^b^ Monitoring Card at Home #2^f^	2013	Binh Thuan provincial DoH	*Maternal*	6	–	–	–	–	–	–	–	–	–	–	–	–	–	–	**340** (94%)	**362**
				*Child*	0	0	0	0	0	0	0	0	12	0	1	0	0	0	13	**22** (6%)	
[16]	MCH^b^ Monitoring Card at Home #3^f^	2013	Thanh Hoa provincial DoH	*Maternal*	6	–	–	–	–	–	–	–	–	–	–	–	–	–	–	**360** (94%)	**381**
				*Child*	0	0	0	0	0	0	0	0	12	0	0	0	0	0	12	**21** (6%)	
[17]	MNH^c^ Monitoring Card at Home #1^g^	[n.a.]	Phu Tan district, An Giang Province	*Maternal*	0	–	–	–	–	–	–	–	–	–	–	–	–	–	–	**318** (98%)	**324**
				*Child*	0	0	0	0	0	0	0	0	0	0	0	0	0	0	0	**6** (2%)	
[18]	MNH^c^ Monitoring Card at Home #2^g^	2013	Thanh Hoa provincial DoH	*Maternal*	0	–	–	–	–	–	–	–	–	–	–	–	–	–	–	**318** (98%)	**324**
				*Child*	0	0	0	0	0	0	0	0	0	0	0	0	0	0	0	**6** (2%)	
[19]	MNH^c^ Monitoring Card at Home #3^g^	2013	Quang Nam provinical DoH	*Maternal*	0	–	–	–	–	–	–	–	–	–	–	–	–	–	–	**348** (97%)	**357**
				*Child*	0	0	0	0	0	0	0	0	0	0	0	0	0	0	0	**9** (3%)	
[20]	MNH^c^ Monitoring Card at Home #4^g^	2013	Hoa Binh provincial DoH	*Maternal*	0	–	–	–	–	–	–	–	–	–	–	–	–	–	–	**318** (98%)	**324**
				*Child*	0	0	0	0	0	0	0	0	0	0	0	0	0	0	0	**6** (2%)	
[21]	MNH^c^ Monitoring Card at Home #5^g^	2013	Ba Ria -Vung Tau provincial DoH	*Maternal*	0	–	–	–	–	–	–	–	–	–	–	–	–	–	–	**397** (98%)	**406**
				*Child*	0	0	0	0	0	0	0	0	0	0	0	0	0	0	0	**9** (2%)	
[22]	Nutrition & Health Care Handbook	2013	National Institute of Nutrition, MoH	*Maternal*	0	–	–	–	–	–	–	–	–	–	–	–	–	–	–	**1** (3%)	**33**
				*Child*	0	0	0	1	0	0	10	11	0	0	19	0	0	0	19	**32** (97%)	
[23]	Health Checkup Handbook	2013	Dept of Medical Services Administration, MoH	*Maternal*	25	–	–	–	–	–	–	–	–	–	–	–	–	–	–	**25** (33%)	**75**
				*Child*	0	0	0	0	0	0	0	0	0	0	0	0	20	30	50	**50** (67%)	

**Total****h**				**Maternal**	**109**	**–**	**–**	**–**	**–**	**–**	**–**	**0**	**0**	**0**	**0**	**0**	**0**	**0**	**0**	**4393** (62%)	**7043**
				***Child***	**10**	**0**	**0**	**31**	**0**	**56**	**263**	**360**	**777**	**0**	**225**	**0**	**304**	**747**	**2053**	**2650** (38%)	

a Categorisation of the items for recording are based on and adapted from Kerber et al. (14).

b MCH: maternal and child health.

c MNH: maternal and newborn health.

d,e,f,g To distinguish each from these home-based records titled the same, numbering was added by the authors.

h The total number of items recording is the simple sum of the number of required items included in the 23 home-based records without considering overlapping.

### Data collection

A structured self-administered questionnaire was sent to the directors of the Provincial Reproductive Healthcare Centers (a technical division responsible for reproductive, newborn, and child health in provincial health departments) of 28 selected provinces, by post in the first weekof July 2013. The questionnaire was composed of 14 questions on HBRs currently available in the 28 provinces (e.g. the number of HBRs currently available and implemented; whether each HBR was nationally scaled-up or province-/district-specific; whether the target users of each HBR were pregnant women/mothers, children, or both pregnant women/mothers, and children; whether each HBR was distributed free of charge; whether the contents of the HBRs were complementary or overlapped). Both telephone- and email-based follow-ups were undertaken to provide the provincial health departments with detailed guidance on the questionnaire and encourage them to complete and submit it by the end of August 2013. The provincial health departments were further requested to send a copy of the HBRs currently available along with the completed questionnaire. Supplementary active collections of copies of ongoing HBRs were conducted, by visiting the four provinces where *MCH Handbook #1* was piloted.

To obtain health workers’ perceptions of and views on the HBR operations and utilizations, 10 semi-structured interviews were conducted at four rural commune health centres in the four provinces where *MCH Handbook #1* was piloted (one commune health centre per pilot province): two interviews in Dien Bien; two in Hoa Binh; three in Thanh Hoa; and three in An Giang. The commune health centres were selected purposively from those located in rural districts at least a 1-hour drive away from the provincial capital. All two or three doctors or nurses responsible for operation of *MCH Handbook #1* and other HBRs at each commune health centre were selected as the interviewees. To obtain the mothers’ perceptions, user-friendliness and ownership of HBRs, 10 semi-structured interviews were conducted in four rural communities located in the catchment areas of the above-selected four commune health centres in the four provinces (one community per pilot province): three interviews in Dien Bien, two in Hoa Binh, three in Thanh Hoa, and two in An Giang. Two or three mothers with children under 12 months of age were selected by snowball sampling. Prior to conducting semi-structured interviews, the methods that would be used for qualitative data processing, along with the study objectives, were shared with health workers and mothers. Open-ended questions were asked using the interview guides, in an anonymous manner to enable interviewees to freely and frankly respond. Each of 20 semi-structured interviews with health workers and mothers lasted for 60–90 min.

### Data analysis

The items for recording were precisely reviewed and counted at the level of data entry columns for all the collected HBRs. All the items for recording identified in the HBRs were further categorised into 40 areas of maternal, newborn, and child health services defined by the Partnership for Maternal, Newborn, and Child Health (13) and later adapted by Kerber et al. (14). The data were entered into and analysed by SPSS for Windows version 22 (IBM/SPSS Inc., Chicago, IL, USA). The semi-structured interviews were transcribed and typed into Microsoft Word 2010 (Microsoft, Redmond, WA, USA). Then, key phrases were coded and categorised for further analyses.

### Ethical consideration

Ethical approval was sought by the authors but it was deemed unnecessary by the Vietnamese MoH, as the MoH officially approved this study as one of several components of the end-line survey for the Project for Implementing Maternal and Child Health Handbook for Nationwide Scaling-up (the Project), an MoH-JICA joint project implemented during the period from February 2011 to December 2014. In addition, the MoH recognised that the level of invasiveness of the study was low enough, as medication, tissue sampling, and blood sampling and asking questions about personal events were included neither in the questionnaire to Provincial Reproductive Healthcare Centers nor in open-ended questions for semi-structured interviews. When conducting semi-structured interviews, informed consent to participate in the study was obtained verbally from health workers and mothers. Since the interviews with health workers were conducted as a part of their duties, written consent was not sought. Considering the illiterate mothers’ reluctance to sign the consent forms, which was likely to derive from feelings of inferiority, written consent was not obtained from them. Piloting of *MCH Handbook #1* was conducted as part of the Project’s activities. The Project was composed of several key activities: piloting *MCH Handbook #1* in the selected four provinces, estimating the impact of *MCH Handbook #1* piloting, estimating the financial requirement for nationwide scaling-up of *MCH Handbook #1*, and making recommendations on strategic directions for nationwide scaling-up of *MCH Handbook #1*.

## Results

### Prevalence of maternal and child health HBRs

There were a total of 23 HBRs for MCH available in the 28 provinces (Table 1). Of the 23 HBRs, four (17%) were exclusively for pregnant women and mothers, whereas five (22%) were exclusively for children. Thirteen (57%) were integrated HBRs for both pregnant women and mothers, and children. *Health Checkup Handbook*, an HBR designed for all age groups of both males and females, was available and applied also to pregnant women, mothers, and children. Of the 23 HBRs, five (22%) were the HBRs nationally scaled-up (i.e. *Child Growth Monitoring Chart*, *Vaccination Card*, *Vaccination Handbook #1*, *Nutrition and Health Care Handbook*, and *Health Checkup Handbook*), whereas the other 18 were either province-specific or district-specific HBRs (Table 1). Although every patient is required to purchase a *Health Checkup Handbook* per episode at their initial visit to governmental hospitals (USD 0.14), all 22 other HBRs were provided to patients free of charge at commune health centres.

**Fig. 1:**
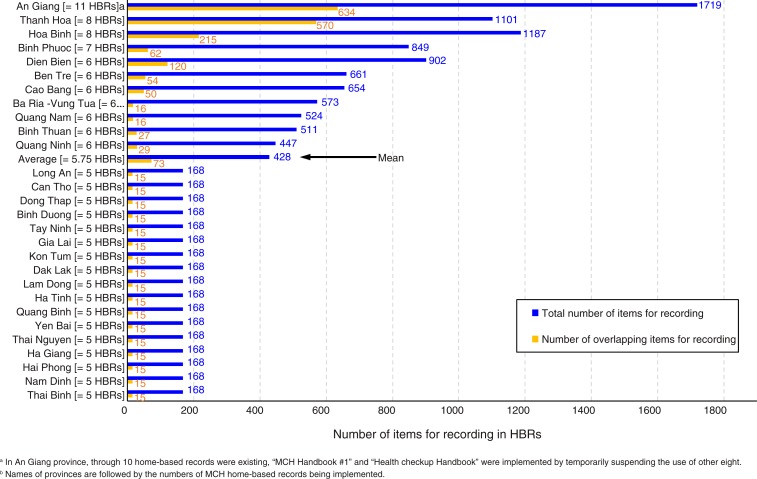
Number of items for recording in home-based records implemented in 28 provinces.

Of the 28 provinces studied, 17 (61%) were implementing only five HBRs which have been already nationally scaled up: *Child Growth Monitoring Chart*, *Vaccination Card*, *Vaccination Handbook #1*, *Nutrition and Health Care Handbook*, and *Health Checkup Handbook* (Table 1). The other 11 provinces (39%) were implementing one or more additional HBRs that were not yet nationally scaled up but were either under piloting for possible nationwide scaling-up or under *ad hoc* province-specific initiatives. The mean number of HBRs implemented in the 28 provinces was 5.75 (Fig. 1). This implies over-availability of HBRs for MCH. Three provinces (11%) implemented at least one of four HBRs developed exclusively for pregnant women (Table 1). All 28 provinces implemented four or five HBRs developed exclusively for children under five years of age. Nine provinces (32%) implemented at least one of thirteen HBRs developed for both pregnant women and mothers, and children (Table 1). Thus, the prevalence rates of HBRs for maternal health, for child health, and for both maternal health and child health in 28 provinces were 14% (4/28), 100% (28/28), and 32% (9/28), respectively.

### Overlapping items for recording between HBRs

**Fig. 2:**
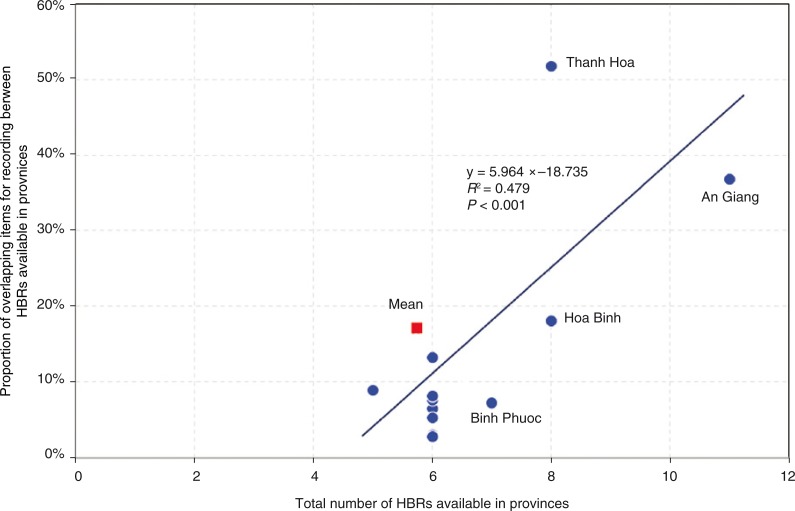
Relationship between the total number of home-based records and the proportion of overlapping items for recording.

Table 2 presents the number of items for recording in each HBR, by area of maternal, newborn, and child health services. The items for recording were precisely counted at the level of data entry columns and were further categorised into 40 areas of maternal, newborn, and child health services. For instance, of 490 items for recording categorised into ‘antenatal care visit ≥4’, 38 were the items for recording for the first antenatal care visit and were composed not only of those related to diagnosis results (e.g. abdomen circumference, blood pressure, presence of oedema, urine protein concentration, and consultation results), but also of those related to clinical administrative procedure (e.g. date of antenatal care visit, date of next appointment, name of health worker, and signature of health worker). Each item for recording was independently counted. *MCH Handbook #3* accommodates entries up to the 18th antenatal care visit, whereas *MCH Handbook #1* covers only eight antenatal care visits. Moreover, the items to be recorded for the first antenatal care visit alone differ from one HBR to another. This great diversity of items for recording and the number of antenatal care visits in the collected HBRs resulted in the identification of 490 items for recording only for the category ‘antenatal care visit ≥4’. The total number of items for recording varied significantly between the HBRs, from four to 854. There were a total of 2,435 items for recording found in 23 HBRs. Of the 2,435 items, 1,171 (48.1%) were found only in a single HBR, while 495 (20.3%) were found in two HBRs (Table 3). This finding implies that 495 items for recording overlapped between two HBRs. Of 1,719 items for recording found in the 11 HBRs available in An Giang province, 634 (37%) overlapped between at least two of the 11 (Fig. 1). Similarly, of 1,101 items for recording found in the eight HBRs available in Thanh Hoa province, 570 (52%) overlapped between at least two of the eight. The greater number of HBRs are implemented, the greater proportion of items for recording overlaps and is commonly included in the HBRs (Fig. 2). The average province of the 28 would have a total of 428 items for recording with 17% overlaps between them, in 5.75 HBRs (Figs. 1 and 2).

**Table 3:** Frequency of overlapping items for recording among 23 home-based records

Number of HBRs in which an item for recording	Number of items for recording (*N=*2435)
1	1,171 (48.1%)
2	495 (20.3%)
3	159 (6.5%)
4	114 (4.7%)
5	85 (3.5%)
6	45 (1.8%)
7	25 (1.0%)
8	240 (9.9%)
9	8 (0.3%)
10	4 (0.2%)
11	17 (0.7%)
12	25 (1.0%)
13	20 (0.8%)
14	6 (0.2%)
15	21 (0.9%)

Total	2,435 (100.0%)

**Fig. 3:**
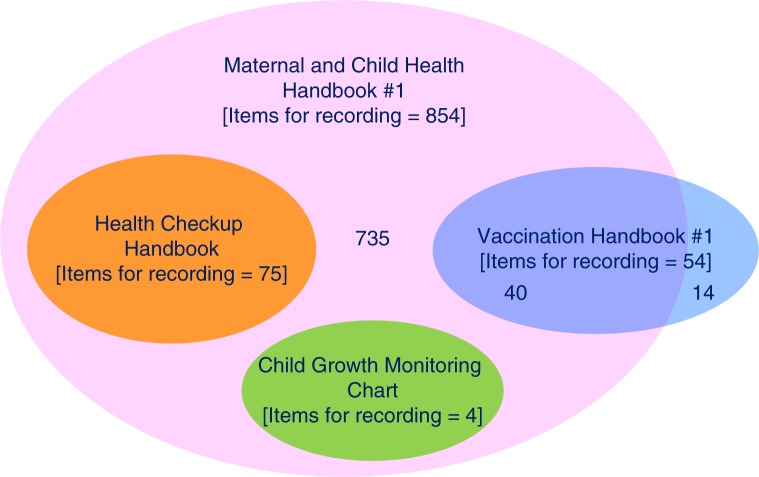
Overlapping items for recording between four major home-based records.

Of all 2,435 items for recording included in the 23 HBRs, 21 were the ones that most commonly overlapped across 15 HBRs (Table 3). Yet, needless to say, not all but only part of the 23 HBRs were implemented in each province. Moreover, five HBRs were implemented as a requirement by the central MoH as the tools that were already nationally scaled up (Table 1). Therefore, to explore the level of overlapping items for recording in HBRs whose implementation was or would be required in all 28 provinces, the subset containment relationship of the items for recording was analysed among three HBRs nationally scaled up and an HBR being piloted for an upcoming nationwide scale-up. The other two of five HBRs already nationally scaled up were excluded from the analysis for two reasons. First, it is a reality that the level of implementation of *Nutrition and Health Care Handbook* was not universal but patchy primarily due to chronic stock-outs of its copies at commune health centres, though it was officially scaled up nationwide. Second, *Vaccination Card* was used commonly as an alternative in a complementary manner, only when *Vaccination Handbook #1* was out of stock at commune health centres. The results of analysis were presented in a Venn diagram (Fig. 3) (15). The 75 and four items for recording in *Child Growth Monitoring Chart* and *Health Checkup Handbook*, respectively, were all included in *MCH Handbook #1*. Of the 54 items for recording in *Vaccination Handbook #1*, 40 (74%) were included in *MCH Handbook #1*. Note that the remaining 14 items for recording (54-40) were related to six-time supplementation of vitamin A, which is not required by the Vietnamese national child immunisation policy (16). Therefore, of the four HBRs analysed in Fig. 3, *MCH Handbook #1* is the only comprehensive HBR that encompassed all 119 minimum required items for recording (75+4+40) commonly found in all three already scaled-up HBRs and national policies/guidelines.

### Users’ perceptions and views on HBRs

Similarly to routine health management information system practice in other developing countries, in Vietnam, it is the health workers’ responsibility to record the data of antenatal check-ups, delivery, postnatal check-ups, child immunisations, and monitoring of child growth, in several facility-based logbooks. They are further required to very carefully transcribe the data into the HBRs mothers bring in, without making data-entry errors. This is necessary not only because more transcriptions must be done manually due to the presence of multiple HBRs, but also because the order and wording of items for recording for the same services significantly or slightly differ between HBRs. Health workers tend to perceive that recording the same data into several HBRs is inefficient and demotivating. For instance, one nurse stated the following:

I sometimes feel ineffective when recording the same data into several HBRs for the same patient without being convinced of a critical need for it … But I do it just because I have to.

At some commune health centres, recording the same data into multiple HBRs was justified by emphasising the need for maintaining knowledge and skills on how to fill out all the ongoing HBRs in case of a stock-out of any scaled-up tools or a phase-out of any piloted tools. Distribution of multiple HBRs to mothers may lead them to mishandle or lose some of them. To avoid unnecessary confusion and loss of HBRs, some health workers gave only one HBR to mothers and filed the rest at the commune health centre as a backup. For instance, one medical doctor stated the following:

We need to be realistic enough to record the same data into even two or three HBRs to keep ourselves skilled at filling out and reading each HBR. Once we stop using one of the HBRs, we may forget its usage and need to learn again about it from scratch. We don't know which HBR will be sustained and which will not, at our level. But if we give mothers two or three HBRs, they tend to lose any of them. We think that giving mothers multiple HBRs free of charge makes them not serious but rather spoiled, as if they can be provided with extra HBRs anytime in the event of their loss.

Implementation of province-specific HBRs is likely to confuse health workers in other provinces, when they are brought in by pregnant women or mothers. For instance, one nurse stated the following:

I know how to record the results of antenatal care services in *MCH Handbook #1*, as it has been piloted in our province for the last couple of years. But I become less comfortable and less confident in recording data into other types of MCH Handbook that are being implemented not in this province but in other provinces. Last month, one pregnant woman, who was back at her parents’ home, brought an HBR with which we were not familiar. It was the HBR being distributed only in a neighbouring province. The method of recording the data was very different from ours. Such a province-specific HBR as this is sometimes brought in by pregnant women who have temporarily come back to this village to give birth in their hometown.

We observed that pregnant women and mothers carefully kept HBRs in a drawer at home. Yet, having multiple HBRs at home discouraged mothers from referring to them by confusing them about which one they should primarily refer to and rely on. They often ended up keeping all the HBRs in a drawer, rarely referring to them, and even treating them roughly. On the other hand, having a single HBR helped mothers focus only on it and more frequently refer to the HBR. Thus, they tended to prefer to receive a more comprehensive HBR (e.g. *MCH Handbook #1*) rather than several fragmented HBRs (e.g. *Antenatal Care Card, Vaccination Handbook #1* and *Child Growth Monitoring Chart*). For instance, one young mother aged 25 stated as follows:

I have both *MCH Handbook #1* and *Vaccination Handbook #1* at home. I did have *Child Growth Monitoring Chart* for my two-year-old boy as well, but lost it somewhere probably a few months ago. But it doesn't matter to me, as the chart is included *in MCH Handbook #1*, as well. My problem is that I have to go back and forth between *MCH Handbook #1* and *Vaccination Handbook #1*, when checking what immunizations my son has already got and needs to get next time. Sometimes the data recorded are different and contradictory between the two handbooks, probably due to health workers’ errors in transcription. That often makes me confused about which handbook to trust …. If there were only one HBR at home, it would be easier for me.

## Discussion

### Need for a geographical integration of HBRs

Of 23 HBRs identified, 17 (74%) were the HBRs that were implemented only in a single province. Implementation of these province-specific HBRs created a great deal of confusion and inconvenience among local health workers. When an HBR developed exclusively for any other province was brought in, health workers felt less comfortable and less confident about recording the results of MCH services. It took longer to record the data in province-specific HBRs, as their format, wording, and types and order of items for recording differed from the HBRs they routinely used. That subsequently could cause data-entry errors and data mistranscription not only between an HBR and facility-based logbooks, but also between the multiple HBRs mothers brought in.

In many countries, including Vietnam, women tend to return to their parents’ home to give birth, which is often far away from their current place of residence (17, 18). In addition, interprovincial migration in Vietnam has been increasingly prevalent, particularly among females (19). Therefore, it is critical to develop a nationally standardised HBR for MCH, in order to ensure accuracy in data recording and transcription regardless of from where pregnant women and mothers visit health facilities. Moreover, province-specific HBRs are likely to be more transient and phase out sooner than nationally standardised ones. This is because their production often depends on *ad hoc* project-based funding (e.g. from NGOs and international development agencies), making the sustainability of province-specific HBRs vulnerable. NGOs and sometimes international development agencies have reportedly suggested provincial and district health departments that project-specific HBRs should be implemented as essential parts of their MCH project activities, without adequate consultation and coordination with the central MoH and provincial health departments, respectively. This method of *ad hoc* HBR implementation only in targeted provinces or districts during the limited lifespan of a project is likely to lead to an increase in the availability of multiple HBRs in certain parts of the country. In particular, in countries such as Vietnam where numerous province-specific HBRs are circulated without integration or coordination, it is an urgent task to develop a nationally standardised HBR for MCH. That will not only help health workers and mothers focus exclusively on one standardised HBR, but will also save HBR operation costs (e.g. HBR production and distribution costs) and thereby increase sustainability.

### Need for a technical integration of HBRs

Of the 23 HBRs identified, nine (39%) had been developed for either maternal or child health exclusively (Table 2). Moreover, of the four HBRs developed for child health, one was designed exclusively for child growth monitoring, while the other three were designed exclusively for child immunisation monitoring. It is expected that fragmentation of HBRs into three technical areas (i.e. antenatal care, child growth monitoring, and child immunisation) neither supports nor promotes a continuum of care for pregnant women, mothers, newborns, and children. Note that an HBR covering all the MCH stages throughout pregnancy, delivery, postnatal period, and childhood provides significant help in ensuring a continuum of seamless maternal, newborn, and child care (20, 21). Moreover, to increase service coverage and efficiency, WHO and UNICEF have been jointly promoting integration of MCH and child immunisation services (22). A systematic review of 32 earlier studies confirmed the increase in delivery of services integrated between MCH and child immunisation (23). In line with the accelerated momentum for service integration, HBRs for MCH should be synchronously integrated into a single HBR, to effectively promote continuous care for pregnant women, mothers, newborns, and children.

A few expanded programme on immunisation (EPI) experts have raised concerns about a potential compromise in user-friendliness due to smaller fonts in the HBRs integrated between immunisation and other child health services (3). Yet, their view is not necessarily supported by earlier studies on users’ dissatisfaction toward integrated HBRs and subsequent poorer recording practices. It is critical to increase the user-friendliness rather of the totality of HBRs for MCH, by integrating the HBRs in order to prevent pregnant women and mothers from having multiple fragmented HBRs. Clearly, it would be of key importance to keep an integrated HBR simple in a well-balanced manner, by including only the minimum number of items required for recording and using an appropriate font size. The levels of user-friendliness and simplicity of integrated HBRs are open to debate, as they tend to include a greater number of items for recording. For instance, *MCH Handbook #1*, the only integrated HBR targeted for nationwide scaling-up, has the greatest number of items for recording (=854) of the 23 HBRs (Table 2). Of the 854 items for recording in *MCH Handbook #1*, 277 (32%) are concerned with postnatal care (41; 5%) or child illnesses and development (236; 27%). Of those 277 items for recording that are essential for monitoring postnatal, infancy, and childhood health status (Table 2), only five and 16 are found in *Vaccination Handbook #1* and *Child Growth Monitoring Chart*, respectively. Moreover, the 239 items to be recorded concerning childhood illnesses and development, which seem at a glance to be too many, are divided into 10 different age-stages: 1st week after birth, 2nd to 6th weeks after birth, 1–3 months of age, 4–6 months of age, 7–9 months of age, 10–12 months of age, 13–19 months of age, 19–23 months of age, 2–3 years of age, and 5–6 years of age. Thus, *MCH Handbook #1* has been designed to enable health workers and mothers to record child health data of 24 items for recording per age-stage (239/10) until a child becomes six years of age. This strategic design keeps mothers encouraged to retain and use *MCH Handbook #1* on a sustainable basis. A great difference in the numbers of items for recording between the HBRs (Table 2) implies only the inadequacy of items required for postnatal, infancy, and childhood healthcare in non-integrated HBRs rather than an excess of items required by the integrated HBRs.

### Coping strategies for multiple HBRs

At the commune health centres in Hoa Binh province, where eight HBRs were available, at least four HBRs were used in practice (i.e. *MCH Handbook #1*, *Vaccination Handbook #1*, *Child Growth Monitoring Chart*, and *Health Checkup Handbook*). Only when *Vaccination Handbook #1* was out of stock, was *Vaccination Card* used in a complementary manner as its proxy tool. To avoid confusion among mothers due to the presence of multiple HBRs at home, health workers gave only *MCH Handbook #1* to them and kept the others as backup at commune health centres. As described by a health worker in a semi-structured interview, there are some risks of possible elimination or disappearance of respective HBRs. Note that no additional HBR copies after stock-out often implies, in real terms, spontaneous termination or elimination of the HBR. The risk of possible HBR termination discourages and prevents health workers at commune health centres from focusing only on *MCH Handbook #1*, while admitting it covers all the necessary items for recording (Fig. 3). This justification for the parallel use of three HBRs (i.e. *MCH Handbook #1*, *Vaccination Handbook #1*, and *Child Growth Monitoring Chart*) makes sense as a practical strategy for hedging the risks of possible termination of any HBRs. In contrast, in An Giang province, where the greatest number of HBRs (=11) were available, out of all the 28 provinces, only *MCH Handbook #1* was used in practice, by temporarily suspending the use of the other 10, to save the opportunity costs of health workers having to record the same data into multiple HBRs and to avoid errors in recording. An Giang Provincial Health Department has been committed to sustaining *MCH Handbook #1*, by even ensuring a line in their budget in the event of termination of the national supplies. This strong commitment effectively enables commune health centres to encourage pregnant women and mothers to focus exclusively on *MCH Handbook #1* as a single HBR for MCH.

### Limitations of the study

This study has limitations in the generalisability of prevalence of HBRs for MCH in Vietnam, as it covered 28 of 64 provinces (44%). Yet, because socio-demographic and geographic diversity was ensured during the process of selecting the 28 provinces, we assume that the 28 selected provinces are reasonably representative. This study did not estimate the level of data recording completeness and data transcription precision in each HBR.

## Conclusions

The mean number of HBRs in 28 provinces (=5.75) indicates over-availability of HBRs. All 119 minimum required items for recording that were found in three different HBRs under nationwide scaling-up were commonly included in *MCH Handbook #1*, which was being piloted in four provinces. Implementation of multiple HBRs is likely to confuse not only health workers by requiring them to record the same data into several HBRs, but also mothers regarding which HBR they should refer to and rely on at home. To enable health workers, pregnant women, and mothers to focus on only one type of HBR, province-specific HBRs for maternal and/or child health need to be nationally standardised. Moreover, to ensure a continuum of maternal, newborn, and child healthcare, the HBRs currently fragmented into different MCH stages (pregnancy, delivery, birth, postnatal period, infancy, and childhood) should be integrated. Standardisation and integration of HBRs will help increase the technical efficiency and financial sustainability of operating HBRs. Note that staying in respective technical silos of immunisation, nutrition, child development, and maternal health (24, 25) discourages integration of HBRs and thereby creates a missed opportunity for policymakers to avoid having overlapping items between the HBRs (Fig. 3).

Earlier studies in a number of countries consistently reported that the use of child vaccination cards, as well as MCH handbooks, contributed to promotion of the utilisation of MCH services (1, 4, 20, 21, 26–28). An international comparison of home-based vaccination cards (2) outlined a global picture of the prevalence of nationally representative child vaccination cards. Yet, the presence of fragmented and overlapping implementations of multiple HBRs at the subnational level has been not addressed but rather overlooked or neglected in those previous studies. This is most likely because the majority of earlier studies relied on Demographic and Health Surveys and Multiple Indicator Cluster Surveys as their data sources, which do not typologise MCH HBRs in detail. Note that improvement in the prevalence of child vaccination cards in 23 countries (2) might have been achieved through over-availability of those HBRs. This study, as the first attempt to estimate the level of in-country fragmentation and overlapping of HBRs, is expected to trigger similar studies in other countries. We strongly suggest that further studies should be conducted to explore optimal HBR implementation approaches and to stimulate deeper discussions about HBR fragmentation and overlap.
